# Occult focal cortical dysplasia may predict poor outcome of surgery for drug-resistant mesial temporal lobe epilepsy

**DOI:** 10.1371/journal.pone.0257678

**Published:** 2021-09-30

**Authors:** Arkadiusz Nowak, Aleksandra Bala

**Affiliations:** 1 Department of Neurosurgery, Medical University of Warsaw, Warsaw, Poland; 2 Faculty of Psychology, University of Warsaw, Warsaw, Poland; Sorbonne Universite UFR de Biologie, FRANCE

## Abstract

**Purpose:**

The results of surgery in patients with mesial temporal lobe epilepsy (MTLE) associated with hippocampal sclerosis (HS) are favorable, with a success rate over 70% following resection. An association of HS with focal cortical dysplasia (FCD) in the temporal lobe is one of the potential causes for poor surgical outcome in MTLE. We aimed to analyzed seizure outcome in a population of MTLE patients and recognize the role of occult FCD in achieving postoperative seizure control.

**Methods:**

We retrospectively analyzed postoperative outcomes for 82 consecutive adult patients with the syndrome of MTLE due to HS, who had no concomitant lesions within temporal lobe in MRI and who underwent surgical treatment in the years 2005–2016, and correlated factors associated with seizure relapse.

**Results:**

At the latest follow-up evaluation after surgery, 59 (72%) were free of disabling seizures (Engel Class I) and 48 (58,5%) had an Engel Class Ia. HS associated with FCD in neocortical structures were noted in 33 patients (40%). Analyzes have shown that dual pathology was the most significant negative predictive factor for Engel class I and Engel class Ia outcome.

**Conclusions:**

The incidence of dual pathology in patients with temporal lobe epilepsy seems to be underestimated. An incomplete epileptogenic zone resection of occult focal temporal dysplasia within temporal lobe is supposed to be the most important negative prognostic factor for seizure freedom after epilepsy surgery in MTLE-HS patients. The study indicates the need to improve diagnostics for other temporal lobe pathologies, despite the typical clinical and radiological picture of MTLE-HS.

## 1. Introduction

Mesial temporal lobe epilepsy (MTLE) associated with hippocampal sclerosis (HS) is the most common focal, drug-resistant epilepsy syndrome requiring surgical treatment [1]. Clinically, MTLE is often regarded as a relatively homogeneous entity affecting adults, with seizures characterized by typical ictal semiology and EEG findings [2]. Medical treatment in patients with MTLE fails in most cases [3]. The results of surgery in such patients are favorable, with a success rate over 70% following resection [4]. However, some patients still fail to improve following surgery. The most common reasons for failure are: insufficient resection of mesial structures, neocortical seizure focus, and temporal contralateral or extratemporal focus [5]. Moreover, hippocampal sclerosis which is the most frequent neuropathologic finding in these patients, may be isolated or associated with a second principal lesion such as a tumor, focal cortical dysplasia (FCD) or vascular malformation. Concomitant presence of second lesion, defined as dual pathology, is another potential cause for surgical failure in MTLE. Malformations of cortical development have been increasingly identified in patients with MTLE [6]. An association of hippocampal sclerosis with cortical dysplasia in the temporal lobe has been discovered as a quite common pathology and has come into the focus of interest. The importance of areas of cortical dysplasia in the neocortical temporal lobe for the epileptogenesis is to be determined. Few reports have considered outcomes in relation to the neuropathologic assessment of neocortical structures. In the present study, the authors analyzed the results of epilepsy surgery in a group of patients with drug-resistant MTLE epilepsy associated with hippocampal sclerosis, who underwent standard resection of the anterior temporal lobe. In particular, we aimed to recognize the role of occult FCD in achieving postoperative seizure control.

## 2. Material and methods

### 2.1. Patient population and preoperative evaluation

A total of 196 patients with intractable temporal lobe epilepsy (TLE) were surgically treated at our institution between 2005 and 2016. All cases were considered to have refractory epilepsy after at least two first-line anti-epileptic drugs failed. A retrospective review of this group identified 104 patients with the syndrome of MTLE due to hippocampal sclerosis (MTLE-HS). In the present study, patients with MTLE-HS were included if they had histopathological diagnosis of HS and postsurgical follow-up of at least 2 years. The study includes both patients who had typical appearance of hippocampal sclerosis on preoperative Magnetic Resonance Imaging (MRI) and those who had a normal MRI (nonlesional MRI). Patients who demonstrated MRI findings suggestive of tumor, FCD, cavernoma or other focal brain lesions in addition to HS were excluded from the study. The overall patients characteristics and epilepsy clinical data were obtained from patients medical records.

All the patients underwent an extensive presurgical evaluation that included assessment of clinical data, a neurologic examination, long-term video-EEG monitoring, high-resolution MRI at 1,5T, and neuropsychological evaluation. The presence of increased hippocampal signal on FLAIR (fluid-attenuated inversion recovery) and T2-weighted images, and/or hippocampal atrophy were considered radiographically proven HS. Functional MRI was performed for presurgical language mapping in only a few cases. Interictal PET (positron emission tomography) scans or SPECT (single-photon emission computerized tomography) were obtained to evaluate for a physiological abnormality in the mesial temporal lobe. Finally, Wada test was also performed in all patients to assess for language dominance and memory impairment. Invasive subdural monitoring was never used.

### 2.2. Surgical procedure and pathological examination

All the clinical data for each patient were then reviewed by a multidisciplinary team (neurosurgeon, epilepsy neurologist, neuroradiologist, and neuropsychologist), and if fulfilled criteria for MTLE associated with hippocampal sclerosis, patients were recommended for surgical therapy.

Surgery was performed under general anesthesia using intravenous propofol as the only anesthetic agent. Intraoperative ECoG was performed in all patients. After opening the dura mater silicon grid electrodes (5x4), with intercontact distances of 10mm were placed on the exposed temporal surface of the cortex. Prior to ECoG recording, the propofol infusion was discontinued and the patient remained on sufentanyl. Monitoring time varied from 10 to 20 min. The preresection ECoG recordings were reviewed for the presence of interictal ECoG spiking or continuous epileptiform discharges (CEDs) of various forms and frequency based on Palmini et al. classification criteria [7]. In all cases tailored surgery consisted of removing the temporal pole, the anterior neocortical lateral cortex, the uncus-entorhinal area, and the hippocampus and parahippocampal gyrus. The extension of the resection of the lateral neocortex varied and was assessed individually in each case based on neuropsychological data, preoperative neurophysiology and intraoperative ECoG testing. In general, our temporal lobe resection included the anterior 3,5–4,5 cm of the temporal neocortex, leaving the superior temporal gyrus in order to preserve eloquent area in the dominant hemisphere based on preoperative Wada testing, as presurgical language mapping in functional MRI was performed in only 6 cases. Functional mapping in an awakened patient was never performed. None of the patients underwent selective amygdalohippocampectomy (SAH). Incidental epileptic activity noted on ECoG after temporal lobe resection, and evaluated as a result of surgical irritation was usually not addressed with enhanced surgical resection. However, persistent spiking that produced a broad spreading field was addressed with further resection in some cases. The decision to extend a resection was based on the total clinical picture as measured by risks/benefits of further resection.

Pathological specimens were sent for analysis in all cases and were evaluated for dentate gyrus alterations and degree of neuronal loss [8]. The temporal lobe was examined for the presence of cortical dysplasia [9].

### 2.3. Patient’s follow-up and statistical analysis

All the patients underwent postoperative MRI at the 3-month follow-up to document the completeness of the mesial structures resection and the extent of temporal lobe resection. Surgical and seizure outcomes were assessed in the outpatient epilepsy clinic at 6 and 12 months and then once a year. Data on seizure frequency were recorded at each visit. Postoperative seizure outcome was graded according to the modified Engel classification [10]. Engel Class I seizure control outcome was considered to be successful treatment, among which the most satisfactory was Engel Class Ia outcome. Engel Class II was considered unsatisfactory outcome, and surgical failures were defined as Engel Class III-IV. Antiepileptic medications were managed at the discretion of the treating epileptologist.

Variables predicting failure of surgical intervention were analyzed. The statistical tests used were the Pearson’s chi square, paired t-test, and Student’s t test for univariate analysis. Kaplan-Meyer survival curves were used to investigate the probability of remaining in Engel Class I and Engel Class Ia outcome. Differences between groups were analyzed with the log-rank test for univariate analysis. Multivariate analysis, using a stepwise logistic regression model and forward selection, were performed to determine the impact of the independent clinico-pathological variables on seizure freedom status. All statistical analyses were performed using Statistica 13.3. A p value<0,05 was considered statistically significant.

All data were fully anonymized. Ethics committee waived the requirement for informed consent.

## 3. Results

### 3.1. Patients characteristics and epilepsy clinical data

82 patients fulfilled all inclusion criteria and were analyzed. There were 43 men and 39 women in the cohort. On admission all patients were in good general and neurological condition. The mean age at the time of surgery was 29,6 +-8,2 years (range 18–51 years), and the age at seizure onset ranged from 7 to 33 years (mean 15,1 +-5,4 years). The mean duration of epilepsy was 14,4 +-7,3 years (range 4 to 34 years). History of initial precipitating injury including febrile seizures, perinathal asphyxia, meningitis, and cranial trauma occurred in 53 patients (64,6%). Second generalization of seizures was noted in 25 cases (30,5%). Preoperative generalized status epilepticus occurred in 6 (7,3%) patients. The mean frequency of seizures before surgery was 9,1 +-5,5 per month (range 1–25).

Preoperative MRI showed typical hippocampal atrophy appearance or/and increased hippocampal signal on FLAIR images in 67 (82%) patients. Preoperative scalp EEG recordings were reviewed retrospectively and searched for the presence of sharp waves or spikes and rhythmic epileptiform discharges (REDs) [11]. EEG recordings demonstrated interictal sharp waves or spikes in the ipsilateral temporal lobe with ipsilateral propagation of spikes in 53 patients, and bilateral propagation in 29 (35,4%) patients. REDs were observed in only 3 (3,7%) patients and were focal temporal in all cases. 46 (56%) patients had a memory asymmetry on Wada testing compatible with mesial temporal lobe dysfunction in the ipsilateral temporal lobe.

### 3.2. Surgical treatment

Forty patients underwent left sided surgery (48,8%) and 42 underwent right sided surgery. 42 (51,2%) patients were operated on the side of the dominant hemisphere. Intraoperative electrocorticography was routinely performed during all surgeries. All patients had sporadic spikes, and 21 had high-amplitude interictal spikes recorded only from mesial structures. CEDs of various forms were observed in 14 patients. Epileptic activity on ECoG recordings after temporal lobe resection was observed in 12 patients. In six patients these discharges were assessed as the result of surgical irritation. In the other 6 patients persistent temporal spiking was addressed with further resection and in three cases it was still present. No patient died during surgery or the postoperative course. Major neurological postoperative complications (excluding hemianopsias) occurred in 5 cases (6,1%): permanent hemiparesis in 1 case (1,2%), temporary dysphasia in 2 patients, and transitory hemiparesis in 2 more cases. Perimetric visual field examination after surgery showed the presence of full hemianopia in 5 (6,1%) patients and quadrantanopsia in another 21 patients (25,6%). Neuropsychological and psychiatric side effects are not addressed in this study. Postoperative seizures were noted in 6 patients (7.3%) within the first week of surgery.

### 3.3. Pathology

Hippocampal sclerosis of varying degrees was detected in all patients. In 25 patients (30%) surgical specimens were not well preserved enough to allow for a correct assessment under the ILAE Classification criteria. Evaluation of hippocampal structures in the remaining specimens revealed the most common HS Type 1 in 46 patients (80,7%), followed by HS Type 2 and HS Type 3, in 9 (15,8%) and 2 (3,5%) patients, respectively. Interestingly, HS associated with FCD type 2 (HS-FCD) in neocortical structures were noted in as many as 33 patients (40,2%) (FCD type 2A and FCD type 2B in 31 and 2 patients, respectively). There were no patients with HS associated with other principal lesions (e.g. tumor). To evaluate the possible clinico-pathologic correlations between the patients with the presence and absence of an associated occult cortical pathology, the cohort was divided into two groups of isolated HS and HS-FCD. Table 1 shows that the patients with HS and FCD type 2 had epilepsy significantly longer (p = 0,014) and were significantly older at time of surgery (p = 0,041) than those with isolated HS (t-Student test). Incidence of CEDs in neocortical ECoG recordings was significantly higher in patients with HS and FCD type 2 (p = 0,0004, Pearson’s chi square test).

**Table 1:** Comparison of isolated HS and HS associated with FCD type 2 patients as to clinical data.

Patient characteristics	Isolated HS	HS-FCD	P Value
	n/N (%) or mean +- SD	n/N (%) or mean +- SD	
Sex, male*	30/49 (61,2)	13/33 (39,4)	0,052
Initial precipitating injury, yes/no *	35/49 (71,4)	18/33 (54,5)	0,117
Age at epilepsy surgery, years **	27,31 +/− 7,442	31,64 +/− 9,027	0,042
Age at epilepsy onset, years**	15,29 +/− 5,708	14,82 +/− 5,157	0,707
Duration of epilepsy, years**	12,80 +/− 5,916	16,82 +/− 8,608	0,014
Seizure frequency per month**	9,14 +/− 5,891	9,12 +/− 5,005	0,986
Secondary generalization of seizures*	17/49 (34,7)	8/33 (24,2)	0,313
REDs displayed on scalp EEG***	0/49	3/33 (9)	0,062
CEDs displayed on intraoperative ECoG***	2/49 (4,1)	12/33 (36,4)	0,0004
Bilateral propagation of spikes on scalp EEG*	20/49 (40,8)	9/33 (27,3)	0,208
Presence of auras*	32/49 (65,3)	22/33 (66,7)	0,899
Normal MRI*	6/49 (12,2)	9/33 (27,3)	0,084
Hippocampal insufficiency (Wada testing)*	30/49 (61,2)	16/33 (48,5)	0,254

*Pearson’s chi square test,

** t Student test,

*** Fisher exact test, HS–hippocampal sclerosis, HS-FCD–hippocampal sclerosis associated with FCD type 2, REDs—rhythmic epileptiform discharges, CEDs—continuous epileptiform discharges.

### 3.4. Seizure outcome

**Fig 1:**
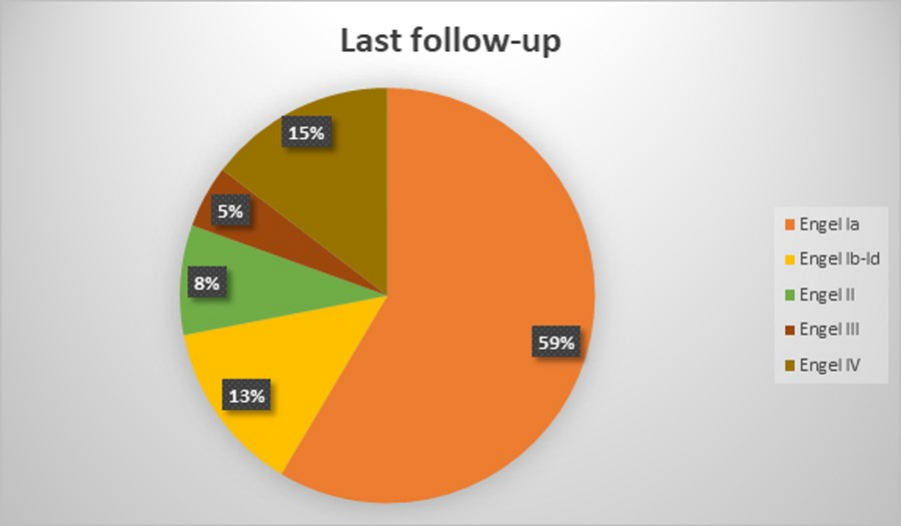
Long-term seizure outcomes. Last follow-up, 82 patients (Engel classification).

**Fig 2:**
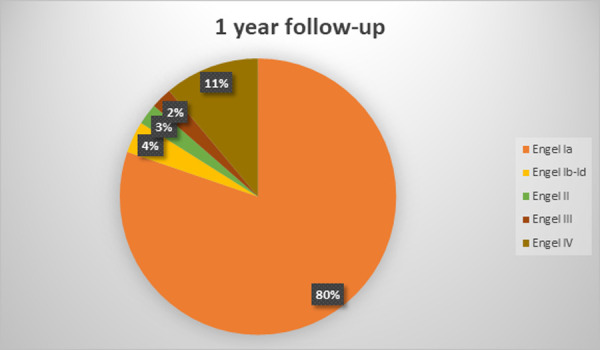
Long-term seizure outcomes. 1 year follow-up, 82 patients (Engel classification).

**Fig 3:**
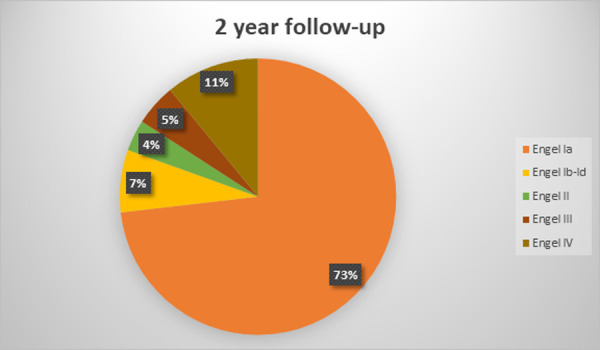
Long-term seizure outcomes. 2 year follow-up, 82 patients (Engel classification).

**Fig 4:**
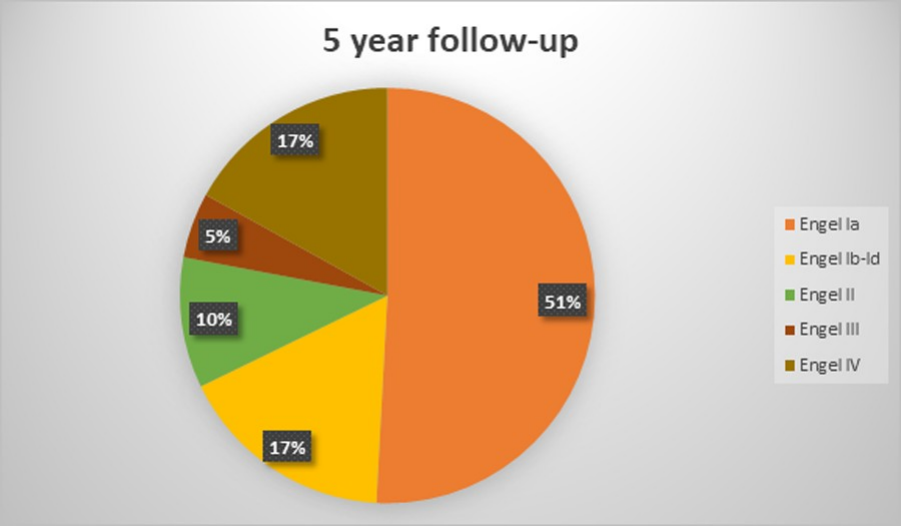
Long-term seizure outcomes. 5 year follow-up, 59 patients (Engel classification).

**Fig 5:**
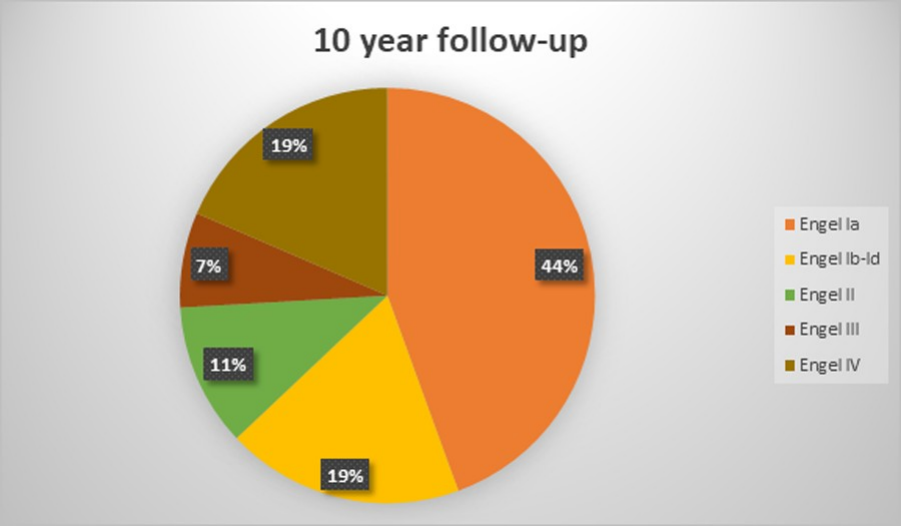
Long-term seizure outcomes. 10 year follow-up, 27 patients (Engel classification).

The mean follow-up duration was 7,6 +-3,6 years (range 2,5–14 years). At the latest follow-up evaluation after surgery, 59 (72%) were free of disabling seizures (Engel Class I) and 48 (58,5%) had an Engel Class IA (excellent result). Among the latter patients, 31 (64,5%) were not taking antiepileptic drugs. 7 (8,5%) patients presented with Engel II outcome (unsatisfactory outcome). 4 patients (5%) were in Engel Class III and 12 (14,5%) had an Engel class IV outcome (surgical failure). Two patients of the cohort died, 3 and 4,5 years after surgery: one from cancer and one from suicide (patient with Engel class IV outcome). Six more patients were lost to follow-up, on average at 4+-3,2 years after surgery (range 2,5–8,5 years). Treatment outcomes varied with the duration of postoperative follow-up and the best results were found in the early postoperative period: at 1-year follow-up 69 (84%) patients were Engel Class I and 13 were Engel Class II-IV. The distribution of outcomes at different follow-up points is depicted in Figs 1–5.

**Fig 6:**
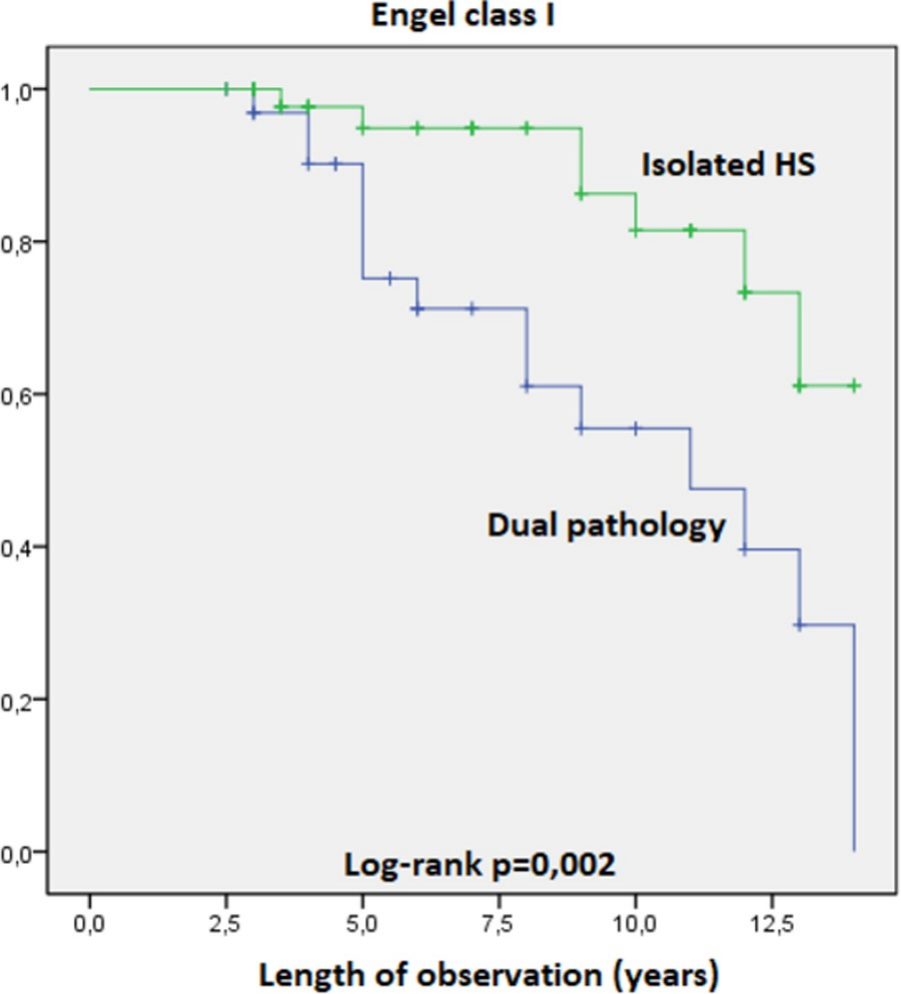
Kaplan-Meier survival curves of the patients in Engel class I by associated pathologies.

**Fig 7:**
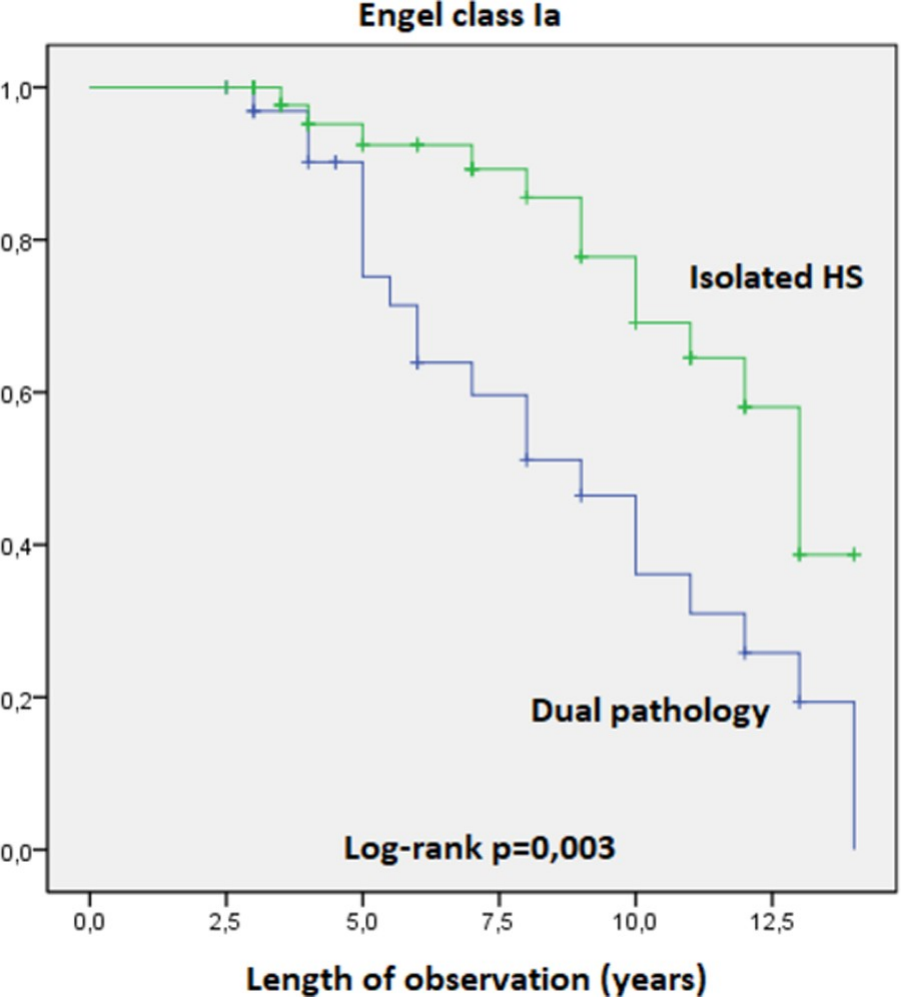
Kaplan-Meier survival curves of patients in Engel class Ia by associated pathologies.

**Fig 8:**
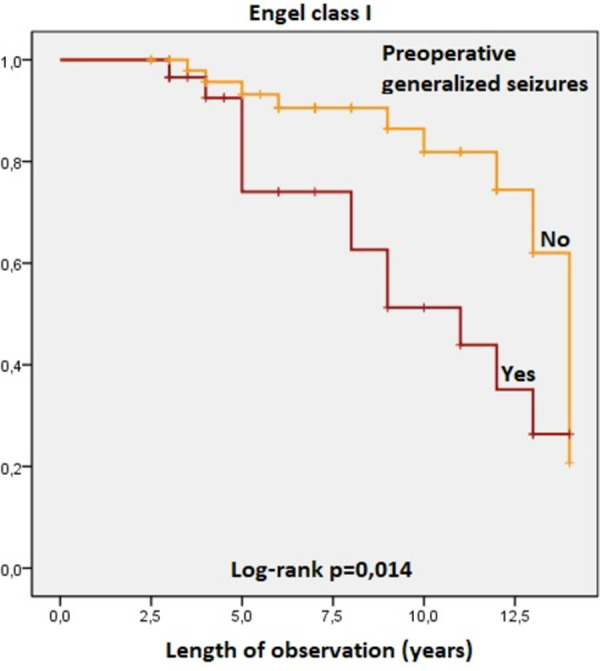
Kaplan-Meier survival curves of patients in Engel class I by seizure semiology.

**Fig 9:**
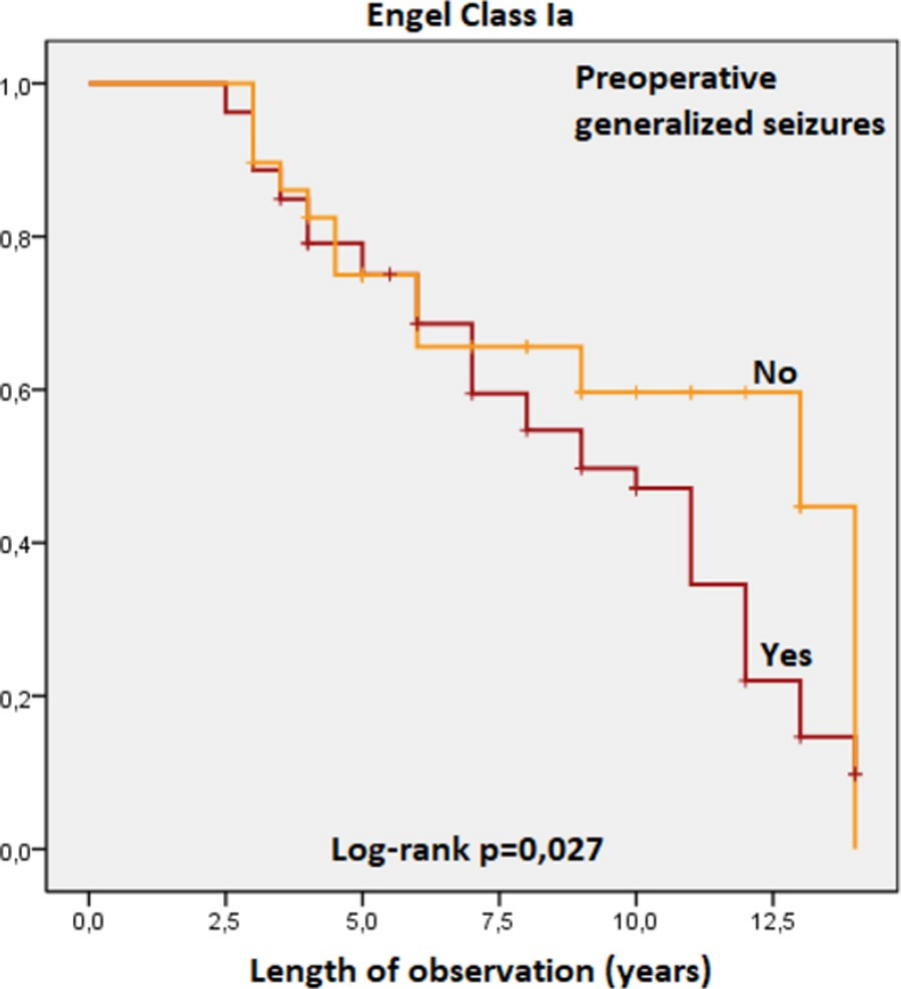
Kaplan-Meier survival curves of patients in Engel class Ia by seizure semiology.

The Kaplan-Meier survival curves revealed statistically significant influence on Engel Class I and Engel class Ia outcomes of the following variables:

1. Patients with dual pathology (HS-FCD) identified on histological examination of temporal lobe samples were significantly less frequently seizure-free compared to patients with no occurrence of FCD associated with HS. Engel class I, log rank test, p = 0,002 (Fig 6). Engel class Ia, log rank test, p = 0,003 (Fig 7).
2. Patients with generalized seizures identified preoperatively had the postoperative seizure-free percentages significantly lower compared to patients without generalization of seizures. Engel class I, log rank test, p = 0,014 (Fig 8). Engel class Ia, log rank test, p = 0,027 (Fig 9).

FCD was detected in 69,6% temporal lobe samples of patients classified as Engel class II-IV at the last follow-up, compared to 28,8% of those who had Engel class I outcome. Table 2 shows that the vast majority of poor treatment outcomes, regardless of the length of postoperative follow-up, were found in patients with dual pathology. It is most evident at 1-year follow-up, when the incidence of the occult FCD in the group of patients with poor seizure outcomes reached 77%.

**Table 2:** The influence of occurrence of the occult FCD on epilepsy surgery outcomes in subsequent years of postoperative follow-up.

Follow-up	The incidence of the occult FCD		
	Engel Class I outcome n/N (%)	Engel Class II-IV outcome n/N (%)	Total n/N (%)
Last*	17/59 (28,8)	16/23 (69,6)	33/82 (40,2)
**1 year**	23/69 (33,3)	10/13 (76,9)	33/82 (40,2)
**2 years**	20/63 (31,7)	13/19 (68,4)	33/82 (40,20
**5 years**	11/40 (27,5)	13/19 (68,4)	24/59 (40,7)
**10 years**	4/17 (23,5)	6/10 (60)	10/27 (37)

The latest follow-up evaluation after surgery (7,6+-3,6 years).

Most clinical data were not significantly discriminative for patients with Engel class I and Engel class Ia outcome. We found that normal mesial temporal lobe defined from MRI was not significantly associated with unfavorable seizure outcome. Detailed information on predictors of seizure outcome is given in Table 3.

**Table 3:** Univariate analysis of predictors of seizure outcome (log-rank test).

Variables	Engel class I outcome		Engel class Ia outcome	
	Average time to event, yr (95%CI)	p	Average time to event, yr (95%CI)	p
**Gender, male/female**	11,9 (10,8–13,0)/ 10,9 (9,4–12,5)	0,469	11,1 (9,9–12,2)/ 9,7 (8,2–11,2)	0,294
**Inicial precipitating injury, yes/no**	11,3 (99,7–12,8)/ 11,6 (10,4–12,7)	0,321	10,3 (9,1–11,5)/ 10,7 (9,3–12,2)	0,711
**Secondary generalization of seizure, yes/no**	9,8 (8,2–11,4)/ 12,4 (11,4–13,4)	0,014	9,1 (7,7–10,6)/ 11,2 (10,1–12,4)	0,027
**Auras, yes/no**	11,2 (10,0–12,4)/ 11,6 (10,1–13,2)	0,305	10,2 (9,0–11,3)/ 10,7 (9,1–12,3)	0,272
**Bilateral propagation of spikes on scalp EEG, yes/no**	11,0 (9,7–12,3)/ 11,9 (10,5–13,2)	0,622	10,1 98,8–11,3)/ 10,9 (9,4–12,3)	0,552
**Normal MRI, yes/no**	11,3 (9,2–13,4)/ 11,5 (10,4–12,5)	0,591	10,3 (9,3–11,4)/ 10,8 (8,9–12,8)	0,053
**Hippocampal insufficiency (Wada testing), yes/no**	12,1 (11,0–13,1)/ 10,7 (9,2–12,2)	0,135	10,9 (9,8–12,1)/ 9,8 (8,4–11,2)	0,217
**Occult FCD associated with HS (Dual pathology), yes/no**	9,9 (8,3–11,5)/ 12,6 (11,6–13,5)	0,002	8,9 (7,4–10,3)/ 11,6 (10,5–12,6)	0,003

A multivariate logistic regression analyses revealed that dual pathology identified on pathologic examination (p = 0,003, Exp(B) = 0,238, 95%CI = 0,078–0,725) was negative predictive factor for Engel class Ia outcome. For Engel class I predictors of unfavorable seizure outcome were preoperative generalized seizures (p = 0,009, Exp(B) = 0,187, 95%CI = 0,053–0,662) and dual pathology (p = 0,001, Exp(B) = 0,114, 95%CI = 0,033–0,393).

12 out of 16 patients who failed epilepsy surgery (Engel Class III and Class IV) underwent comprehensive evaluation to select candidates for reoperation. Two patients refused to reassess their epilepsy and another two patients were lost to follow-up. Only four patients were finally reoperated. The interval between the first and second resections was 3, 3.5, 5 and 7 years. The remaining 8 patients were not qualified for repeated surgery: 2 patients were found to have ictal epileptic activity in the contralateral hemisphere and 6 patients had seizures arising on the side of surgery but originating in the extratemporal location or localized in the posterior temporal region. 3 out of 4 reoperated patients were previously diagnosed with HS in association with lateral temporal dysplasia. The second procedures in these cases included more extensive lateral temporal resection in all patients and resection of residual mesial structures in one patient. The seizure outcome after reoperation was as follows: two patients were Engel Class II, one patient was Class III and one remained Class IV.

## 4. Discussion

The effectiveness, efficacy, and safety of epilepsy surgery for MTLE have been established through one randomized controlled trial [12] and numerous reports [6, 13–15]. Our study on a homogenous population of 82 MTLE-HS patients undergoing surgery showed 72% patients to be Engel class I at their latest follow-up evaluation. These results are in line with other similar series, in which over 2/3 of patients were noted to be free of disabling seizures after surgery [16, 17]. In the remaining group, seizures usually recur during the first two postoperative years, although in accordance with our study, the percentage of patients who remain seizure free decline with longer follow-up [18–20]. An understanding of the factors influencing the outcome following surgery in these patients would allow for a better selection of the correct cohort for intervention and could modify the extent of temporal lobe resection. Most published studies aimed at identifying the factors predicting outcomes analyzed clinical, radiologic and surgical parameters, but it has been pointed that etiology plays a major role in influencing the long-term efficacy of temporal lobe epilepsy (TLE) surgery [14]. Recent studies focused mainly on the histopathological abnormalities within affected hippocampus which are believed to be the main factors predictive of seizure outcome [6, 21–23]. The scope of our interests were mainly histopathological changes in the lateral cortex of the temporal lobe. We have reviewed our series of patients with drug-resistant, surgically treated MTLE, trying to identify predictors of poor surgical outcome and to correlate seizure outcome with the occurrence of occult FCD.

A preoperative generalized tonic-clonic seizures was linked to less favorable seizure outcomes after surgery in accordance with the literature [24–26], although age at time of surgery for was not positive predictive factor. These results stress an early consideration of epilepsy surgery in drug-resistant temporal lobe epilepsy. Of interest, the outcome in MRI-negative HS patients was not significantly worse compared to lesional patients. This indicates the need for early referral of patients with medically refractory epilepsy to an epilepsy center for surgical evaluation despite non-lesional MRI. The most important cause of failure seems to lie, however, in the pathological substrate of this group of patients. Our study addressed concomitant pathologies of neocortical temporal lobe due to the fact that all patients in our cohort underwent anterior temporal lobectomy with various extend of temporal resection. Extrahippocampal pathology, namely FCD, in the temporal lobe, without any MRI imaging findings suggestive of developmental abnormality was noted in as many as 40% in the present series. This exceeds considerably frequency of dual pathology reported so far [27]. Dual pathology refers to the coexistence of mesial temporal sclerosis and extrahippocampal lesion. It is not clear to us why so many patients with histopathologically confirmed MTLE-HS were found to have FCD in the lateral temporal lobe. A partial explanation for this fact may be the use of standard anterior temporal lobe resection in all patients, which allows for a thorough pathological assessment of cortical developmental changes of the temporal neocortex. It can be assumed that in some cases when selective amygdalo-hippocampectomy or limited temporal lobe resection is performed, clinically silent and invisible FCD foci remain undiagnosed. Moreover, it notes that when preparing this analysis, the pathological specimens of the temporal lobe were re-evaluated in accordance with the current classification of cortical dysplasia, and the present incidence of FCD was significantly higher than initially diagnosed. The clinical significance of FCD in temporal neocortex found in patients with HS is an issue if intense debate. A preponderance of hippocampal seizure onset does not exclude an additional extrahippocampal seizure onset zone. It has been reported earlier in literature that in the presence of dual pathology, resection of only the extra hippocampal lesion or only the hippocampus can result in poor outcome. Li et al. [28] demonstrate that in a group of patients with extrahippocampal lesions (including cortical dysgenesis, tumors, contusions, infarcts and vascular malformations) associated with hippocampal atrophy, even if atrophied hippocampus appeared to be the most epileptogenic structure, only 20% of the patients became seizure free after hippocampal removal. In our opinion, the main cause of failure in the treatment of MTLE-HS are occult neocortical foci of FCD in the temporal lobe or extra-temporal FCD. It would seem that when performing non-selective resection, areas of FCD should be removed by anterior temporal lobectomy, and FCD associated with HS should not result in surgical failure. Our observations show that in most cases, the resection of the FCD area is incomplete and the lesion extends beyond the limits of safe resection and even beyond the temporal lobe. Hence, surgical treatment in these cases is often ineffective. Hennesy et al. found that in patients with HS recurrent seizures did not arised from residual hippocampus but relapse came from the temporal neocortex and the ipsilateral and contralateral frontal lobes which suggests that epileptogenesis may be related to occult cortical dysplasia in these locations [29]. In their experience reoperations were considered possible in a small number of patients which is in line with our findings. The primary existence or development of secondary epileptogenic focus during the course of epilepsy is also emphasized by reports in which the rate of dual pathology is low. Mathon et al. found that preoperative history of status epilepticus was associated with a worse surgical outcome which was attributed to cortical lesions induced by the severe course of seizures [30]. In our series, none of the patients with FCD on histopathological examination of the resected specimens demonstrated any extrahippocampal lesion in MR imaging. On the other hand we analyzed in the paper only cases in which the MR images did not suggest any additional focal changes apart from the features of HS. Besides, it is known that even in patients with cortcal dysplasia recognised on MRI the actual area of the lesion is more widespread than that seen on MRI [31]. In most patients with FCD Type IIa T1-weighted and T2-FLAIR 1,5 T MR images are reported normal and do not reveal definitely abnormal signal intensities. Increased subcortical white matter signal is almost exclusive to type FCD Type IIb [9]. In our study, the vast majority of FCD type IIa cases were diagnosed. This, in addition to using 1,5T MRI could explain the lack of visible changes in MRI. The use of 3T and 7T helps to improve the selection of candidates for epilepsy surgery. 3T MRI has been found to improve detection of FCD Type 2 owing to better visualization of the transmantle sign. According to Mallerio et al. 3T MRI was able to clarify equivocal features shown on 1,5T MRI [32]. High field imaging could increase sensitivity for the detection of FCD, however, multimodal 3T MRI analysis that combines morphometry with metrics interrogating tissue intensity, microstructure and function has the ability to dissociate FCD subtypes and predict FCD subtypes with equal sensitivity of 85% [33]. Application of ultra-high field 7T MRI increases the sensitivity to detect an epileptogenic lesion. Van Lanen et al. [34] reported in a systematic review pooled diagnostic gain of 7T MRI over conventional clinical MRI. In this study 31% of included patients with negative 3T had positive 7T MRI and most of them had radiological hallmarks of FCD. FDG-PET data coregistered with MRI data is another valuable tool for the accurate localization of cortical dysplasia, especially in cases of FCD type 2 [35]. Coregistration improves the sensitivity of MRI and PET analyses, hence PET-MRI is invaluable in MRI-negative patients and helps to delineate FCD Type 2 in MRI-positive patients [36]. Small temporal pole encephalocele is another occult temporal lesion, that is hardly recognizable and may lead to surgical failure in temporal lobe epilepsy [37]. Encephalocele is a small herniation of the temporal pole through defect of the skull and meninges and might be the cause of MRI negative and PET positive drug-resistant temporal lobe epilepsy. In these patients initial MRI is often considered normal because the lesion is frequently isointense with the gray matter and skull abnormalities are difficult to identify with MRI [38]. In our opinion intraoperative ECoG may by useful to rule out occult focal cortical dysplasia in patients with MTLE-HS, which is consistent with earlier reports [39]. Currently, high frequency oscillations (HFOs) seem to be better biomarkers for epileptogenic tissue than spikes and can be used for delineating the boundary between the epileptogenic zone and the irritative post-surgical zone not necessary to resect [40]. However, due to the limited availability of the HPOs expensive technology, the maximum safe resection of the temporal lobe can be performed, guided by the ECoG recordings [41]. We think that improvement of outcomes in the temporal epilepsy surgery could be achieved in the mandatory use of high-resolution 3T MRI to improve visualization of possible epileptic substrate (e.g. residual FCD) and more extensive presurgical neurophysiological evaluation to rule out extrahippocampal or extratemporal epileptogenic foci despite the typical clinico-radiological picture of MTLE-HS. The additional benefits of PET-MRI encourage its routine use. Intraoperatively, when the presence of preoperatively undiagnosed FCD foci in the temporal neocortex based on ECoG is suspected, maximum safe resection of the temporal lobe should be performed guided by the ECoG recordings or further awake surgery can be considered.

In the present study recurrent seizures were mostly regarded as arising outside the range of previous resection, extratemporal or contralateral to the previous surgery site. Hence reoperations were considered possible in a small number of patients. After initial surgical failure repeated surgery should be contemplated if recurrent seizures originate from the previous resection site. Diagnostic effort should focus on visualization of epileptic substrate left behind during the first surgery, as patients with visualized lesions benefit the most from reoperations [42, 43]. If residual abnormal cortex is found in the lateral temporal lobe and there are no functional limitations further surgery should be attempted. However, our experience confirms earlier reports that patients with HS associated with FCD are less likely to improve after repeated surgery [43].

### 4.1. Limitations

There may be some possible limitations in this study. This is retrospective, single center study, with lack of randomization. The study group is relatively small and the period of postoperative observation is short. Hence the results after 5 years of observation are only indicative. Unfortunatelly, volumetric studies (preoperative analysis of the temporal lobes and postoperative resection assessment) were not routinely performed in these patients. Moreover, our cohort was evaluated using 1,5 T MRI which could have affected some percentage of FCDs with a normal temporal lobe image. The preponderance of dual pathology in our “nonlesional” cohort-group needs future validation using higher resolution imaging, including an attempt to define the extent of temporal lobe pathology.

## 5. Conclusions

The actual incidence of dual pathology in patients with temporal lobe epilepsy seems to be underestimated. The lack of focal changes in the temporal neocortex on preoperative MRI does not exclude concomitant temporal pathology that may affect the outcome of treatment. An incomplete epileptogenic zone resection of the occult FCD within temporal lobe is supposed to be one of the most important negative prognostic factors for seizure freedom after epilepsy surgery in MTLE-HS patients. The study indicates the need to improve diagnostics for other temporal lobe pathologies, despite the typical clinical and radiological picture of MTLE-HS. It appears that intraoperative ECoG may be used to guide surgical resection of the dysplastic cortex non-visible in the MRI to achieve better seizure control.

## Supporting information

S1 Table Preoperative investigations and intraoperative ECoG. (DOCX)

S2 Table Pathology, postoperative follow-up, and outcome. (DOCX)
