# Supplementary material for: Occult focal cortical dysplasia may predict poor outcome of surgery for drug-resistant mesial temporal lobe epilepsy
Source: PLoS One. 2021 Sep 30;16(9):e0257678. doi: 10.1371/journal.pone.0257678 (PMC8483375; doi:10.1371/journal.pone.0257678)
Supplement: S1 Table — (DOCX) [file pone.0257678.s001.docx]

S1 Table. Preoperative investigations and intraoperative ECoG.

| **Patient** | **Sex; Age at epilepsy onset (yr); Pre-op duration of epilepsy (yr); Age at surgery (yr)** | **Initial precipitating injury** | **Pre-op seizure frequency (per month); Seizure semiology** | **Neurophysiology (scalp EEG, Wada testing)** | **Preresection ECoG** | **Postresection ECoG** |
| --- | --- | --- | --- | --- | --- | --- |
| 1 | male; 8; 24; 32 | febrile seizures | 6; no auras; SGTC, no SE | BPS; IDH | SS | normal |
| 2 | male; 13; 5; 18 | none | 2; no auras; no SGTC, no SE | IPS; ITLD; IDH | SS; HAIS | normal |
| 3 | male; 31; 14; 45 | cranial trauma | 8; auras, no SGTC, no SE | IPS | SS | normal |
| 4 | female; 20; 31; 51 | febrile seizures | 16; auras, SGTC; no SE | IPS | SS; CEDs | EA |
| 5 | female; 13; 5; 18 | febrile seizures | 4, no auras, no SGTC, no SE | IPS; ITLD; IDH | SS; HAIS | normal |
| 6 | male; 18; 13; 31 | none | 25; no auras; no SGTC, no SE | IPS | SS | normal |
| 7 | male; 12; 28; 40 | febrile seizures | 2; auras; SGTC; SE | BPS; ITLD; IDH | SS; HAIS | normal |
| 8 | female; 17; 6; 23 | none | 12; auras; SGTC; no SE | IPS; REDs; ITLD | SS; CEDs | normal |
| 9 | female; 15; 14; 29 | febrile seizures | 20; auras, no SGTC; no SE | IPS | SS; HAIS | normal |
| 10 | female; 15; 10; 25 | febrile seizures | 16; no auras, no SGTC, no SE | IPS; ITLD; IDH | SS | EA; FR |
| 11 | male; 14; 14; 28 | none | 20; auras; SGTC, SE | IPS; IDH | SS | normal |
| 12 | female; 18; 31; 49 | perinathal asphyxia | 6; auras; no SGTC; no SE | IPS | SS; CEDs | normal |
| 13 | male; 14; 12; 26 | febrile seizures | 2; auras; no SGTC, no SE | BPS; IDH | SS; HAIS | normal |
| 14 | female; 7; 24; 31 | febrile seizures | 8; no auras; no SGTC; no SE | BPS; ITLD | SS | normal |
| 15 | female, 19; 7; 26 | none | 20; no auras; SGTC, no SE | IPS | SS | EA |
| 16 | female; 22; 7; 29 | meningitis | 8; no auras; no SGTC; no SE | BPS; ITLD, IDH | SS; HAIS | normal |
| 17 | male; 28; 15; 43 | cranial trauma | 8; auras; no SGTC; no SE | IPS; ITLD | SS | normal |
| 18 | male; 17; 19; 38 | none | 12; no auras; SGTC, SE | IPS; ITLD; IDH | SS; CEDs | normal |
| 19 | male; 17; 21; 38 | febrile seizures | 8; auras; no SGTC; no SE | IPS | SS; CEDs | normal |
| 20 | female; 23; 13; 36 | none | 12; auras; SGTC; no SE | IPS; ITLD; IDH | SS | normal |
| 21 | male; 24; 20; 44 | febrile seizures | 2; auras; no SGTC; no SE | IPS; ITLD; IDH | SS; HAIS | normal |
| 22 | male; 13; 30; 43 | none | 15; auras; no SGTC; no SE | IPS; IDH | SS | EA; FR; EAFR |
| 23 | female; 27; 2; 29 | cranial trauma | 20; no auras; SGTC; no SE | BPS; IDH | SS | normal |
| 24 | male; 20; 10; 30 | none | 4; auras; no SGTC; no SE | BPS; ITLD | SS; HAIS | normal |
| 25 | male; 17; 18; 35 | febrile seizures | 12; no auras; SGTC; no SE | IPS | SS | EA |
| 26 | male; 12; 12; 24 | febrile seizures | 8; auras; no SGTC; no SE | IPS; ITLD; IDH | SS | normal |
| 27 | female; 15; 19; 34 | none | 12; auras; no SGTC; no SE | IPS; IDH | SS; HAIS | normal |
| 28 | female; 15; 30; 45 | febrile seizures | 12; no auras; no SGTC; no SE | IPS; IDH | SS | normal |
| 29 | male; 13; 17; 30 | perinathal asphyxia | 16; auras; SGTC, SE | IPS; ITLD | SS | normal |
| 30 | female; 9; 18; 27 | none | 2; auras; no SGTC; no SE | IPS; ITLD, IDH | SS | normal |
| 31 | male; 13; 9; 22 | febrile seizures | 20; auras; SGTC, no SE | BPS | SS | normal |
| 32 | male; 11; 12; 23 | febrile seizures | 16; no auras; no SGTC, no SE | IPS; REDs; ITLD; IDH | SS; CEDs | EA; FR |
| 33 | female; 12; 20; 32 | febrile seizures | 4; no auras; no SGTC; no SE | BPS | SS; HAIS | normal |
| 34 | female; 9; 22; 31 | none | 2; auras; no SGTC; no SE | IPS; ITLD | SS | normal |
| 35 | male; 20; 8; 28 | febrile seizures | 8; no auras; SGTC; no SE | IPS; IDH | SS | normal |
| 36 | male; 14; 18; 32 | cranial trauma | 12; auras; no SGTC; no SE | BPS; ITLD | SS; HAIS | normal |
| 37 | male; 12; 9; 21 | febrile seizures | 8; auras; no SGTC; no SE | IPS; ITLD; IDH | SS | normal |
| 38 | female; 19; 4; 23 | none | 8; auras; no SGTC; no SE | IPS; ITLD | SS | normal |
| 39 | female; 10; 27; 37 | febrile seizures | 12; no auras; SGTC; no SE | IPS; ITLD; IDH | SS | EA |
| 40 | male; 9; 9; 18 | none | 16; auras; no SGTC; no SE | BPS | SS | normal |
| 41 | female; 18; 6; 24 | febrile seizures | 8; no auras; no SGTC; no SE | IPS; ITLD | SS; CEDs | normal |
| 42 | female; 12; 13; 25 | febrile seizures | 2; auras; SGTC; no SE | IPS; ITLD; IDH | SS; HAIS | normal |
| 43 | male; 25; 7; 32 | perinathal asphyxia | 8; no auras; no SGTC; no SE | IPS | SS | normal |
| 44 | male; 9; 9; 18 | none | 12; auras; no SGTC; no SE | BPS | SS; CEDs | EA; FR; EAFR |
| 45 | female; 8; 15; 23 | febrile seizures | 6; auras; no SGTC; no SE | BPS; ITLD; IDH | SS | normal |
| 46 | male; 8; 14; 22 | none | 8; no auras; SGTC; SE | IPS | SS | normal |
| 47 | male; 9; 18; 27 | febrile seizures | 12; auras; no SGTC; no SE | BPS; ITLD; IDH | SS | normal |
| 48 | female; 10; 12; 22 | febrile seizures | 10; no auras; no SGTC; no SE | BPS; IDH | SS | normal |
| 49 | female; 11; 35; 46 | none | 8; auras; SGTC; SE | IPS; ITLD | SS; HAIS | normal |
| 50 | female; 11; 7; 18 | febrile seizures | 4; auras; no SGTC; no SE | IPS; IDH | SS | normal |
| 51 | male; 12; 13; 25 | none | 6; no auras; no SGTC; no SE | BPS; ITLD | SS; CEDs | normal |
| 52 | male; 12; 8; 20 | febrile seizures | 2; auras; SGTC; no SE | BPS; ITLD; IDH | SS; HAIS | normal |
| 53 | female; 12; 28; 40 | febrile seizures | 10; auras; no SGTC; no SE | IPS | SS | normal |
| 54 | male; 12; 10; 22 | febrile seizures | 14; auras; no SGTC; no SE | IPS; ITLD; IDH | SS | normal |
| 55 | female; 12; 16; 28 | none | 1; auras; no SGTC; no SE | IPS; ITLD | SS; CEDs | EA; FR |
| 56 | female; 13; 17; 30 | febrile seizures | 6; no auras; no SGTC; no SE | BPS | SS | normal |
| 57 | male; 14; 14; 28 | febrile seizures | 4; auras; no SGTC; no SE | IPS; ITLD; IDH | SS; HAIS | normal |
| 58 | male; 14; 16; 30 | none | 2; auras; no SGTC; no SE | IPS | SS; CEDs | normal |
| 59 | female; 12; 26; 38 | febrile seizures | 10; auras; SGTC; no SE | BPS; ITLD | SS | normal |
| 60 | male; 14; 16; 30 | none | 8; auras; no SGTC; no SE | BPS; ITLD; IDH | SS | normal |
| 61 | female; 14; 17; 31 | febrile seizures | 12; no auras; no SGTC; no SE | IPS; ITLD | SS | normal |
| 62 | male; 13; 5; 18 | none | 1; auras; SGTC; no SE | IPS; IDH | SS | normal |
| 63 | male; 15; 10; 25 | febrile seizures | 4; auras; no SGTC; no SE | BPS; ITLD; IDH | SS; HAIS | normal |
| 64 | male; 14; 20; 34 | febrile seizures | 2; no auras; no SGTC; no SE | IPS | SS | normal |
| 65 | male; 15; 15; 30 | febrile seizures | 6; auras; SGTC; no SE | BPS; ITLD; IDH | SS | normal |
| 66 | female; 11; 7; 18 | none | 15; no auras; no SGTC; no SE | IPS; ITLD | SS; HAIS | normal |
| 67 | male; 18; 12; 30 | cranial trauma | 12; auras; no SGTC; no SE | BPS; ITLD; IDH | SS | normal |
| 68 | female; 14; 12; 26 | none | 8; auras; no SGTC; no SE | IPS | SS; CEDs | EA |
| 69 | female; 15; 4; 19 | febrile seizures | 2; auras; no SGTC; no SE | IPS; ITLD | SS | normal |
| 70 | female; 24; 15; 39 | febrile seizures | 4; auras; no SGTC; no SE | BPS; IDH | SS | normal |
| 71 | male; 14; 5; 19 | none | 2; auras; SGTC; no SE | IPS; ITLD; IDH | SS; HAIS | normal |
| 72 | female; 28; 10; 38 | cranial trauma | 12; no auras; no SGTC; no SE | IPS; ITLD | SS | normal |
| 73 | female; 22; 13; 35 | febrile seizures | 20; auras; no SGTC; no SE | IPS; IDH | SS; CEDs | EA; FR; EAFR |
| 74 | male; 33; 15; 48 | meningitis | 4; auras; no SGTC; no SE | BPS; ITLD; IDH | SS | normal |
| 75 | male; 9; 19; 28 | febrile seizures | 16; auras; SGTC; no SE | BPS; ITLD | SS; HAIS | normal |
| 76 | female; 18; 16; 34 | none | 6; auras; no SGTC; no SE | IPS; ITLD; IDH | SS | EA |
| 77 | male; 12; 10; 22 | none | 8; auras; SGTC; no SE | BPS | SS | normal |
| 78 | female; 11; 14; 25 | febrile seizures | 10; auras; no SGTC; no SE | IPS; ITLD | SS; CEDs | normal |
| 79 | female; 20; 11; 31 | none | 6; no auras; no SGTC; no SE | IPS; REDs; IDH | SS | normal |
| 80 | male; 12; 11; 23 | none | 8; no auras; SGTC, no SE | BPS; IDH | SS; HAIS | normal |
| 81 | female; 13; 12; 25 | febrile seizures | 12; auras; no SGTC; no SE | BPS | SS; HAIS | normal |
| 82 | male; 10; 11; 21 | febrile seizures | 10;; auras; no SGTC; no SE | IPS; ITLD; IDH | SS | normal |

yr – years; pre-op – preoperative; SGTC – secondarily generalized tonic-clonic; SE – status epilepticus; IPS – ipsilateral propagation of spikes; BPS – bilateral propagation of spikes; ITLD – ipsilateral temporal lobe dysfunction (hippocampal insufficiency); IDH – ipsilateral dominant hemisphere; REDs – rhythmic epileptiform discharges; CEDs – continuous epileptiform discharges; SS – sporadic spikes; HAIS – high amplitude interictal spikes only from mesial structures; EA – epileptic activity; FR – further resection; EAFR – epileptic activity after further resection
