# Supplementary material for: Occult focal cortical dysplasia may predict poor outcome of surgery for drug-resistant mesial temporal lobe epilepsy
Source: PLoS One. 2021 Sep 30;16(9):e0257678. doi: 10.1371/journal.pone.0257678 (PMC8483375; doi:10.1371/journal.pone.0257678)
Supplement: S2 Table — (DOCX) [file pone.0257678.s002.docx]

S2 Table. Pathology, postoperative follow-up, and outcome.

| **Patient** | **Side of surgery** | **Postoperative complications** | **Histopathology** | **Outcome** | **Notes** |
| --- | --- | --- | --- | --- | --- |
| 1 | left | none | HS type 1; FCD IIA | Engel Class IV | refused reassessment; follow-up 14 yr |
| 2 | left | quadrantopsia | HS type 2 | Engel Class IA | follow-up 14 yr |
| 3 | right | none | HS UAC; FCD IIA | Engel Class IA | follow-up 6 yr |
| 4 | right | EPS | HS type 1; FCD IIA | Engel Class IV | not qualified for repeated surgery; follow-up 14 yr |
| 5 | left | quadrantopsia | HS type 1 | Engel Class IA | follow-up 14 yr |
| 6 | right | none | HS UAC | Engel Class IA | follow-up 13 yr |
| 7 | left | none | HS type 1 | Engel Class II | follow-up 13 yr |
| 8 | right | none | HS UAC; FCD IIA | Engel Class IV | not qualified for repeated surgery; follow-up 13 yr |
| 9 | right | permanent hemiparesis; full hemianopia | HS type 2 | Engel Class IA | follow-up 13 yr |
| 10 | left | quadrantopsia | HS type 1; FCD IIB | Engel Class IA | follow-up 13 yr |
| 11 | left | none | HS UAC | Engel Class IB | follow-up 13 yr |
| 12 | right | quadrantopsia | HS type 1; FCD IIA | Engel Class ID | follow-up 5,5 yr |
| 13 | left | none | HS type 1 | Engel Class IA | follow-up 12 yr |
| 14 | right | none | HS UAC; FCD IIA | Engel Class IA | follow-up 12 yr |
| 15 | right | none | HS type 2; FCD IIA | Engel Class III | not qualified for repeated surgery; follow-up 5 yr |
| 16 | left | none | HS type 1 | Engel Class IA | follow-up 12 yr |
| 17 | right | none | HS UAC | Engel Class IA | follow-up 12 yr |
| 18 | left | full hemianopia; EPS | HS type 1 | Engel Class II | follow-up 12 yr |
| 19 | right | none | HS type 1; FCD IIA | Engel Class IV | refused reassessment; follow-up 12 yr |
| 20 | left | quadrantopsia | HS UAC | Engel Class IC | follow-up 11 yr |
| 21 | left | none | HS type 1 | Engel Class IA | follow-up 11 yr |
| 22 | right | quadrantopsia | HS type 1; FCD IIA | Engel Class II | follow-up 11 yr |
| 23 | left | none | HS type 2 | Engel Class IA | follow-up 11 yr |
| 24 | right | quadrantopsia | HS type 1; FCD IIA | Engel Class IA | follow-up 4,5 yr |
| 25 | right | none | HS type 1 | Engel Class IA | follow-up 11 yr |
| 26 | left | none | HS type 1 | Engel Class IA | follow-up 11 yr |
| 27 | left | none | HS UAC; FCD IIA | Engel Class IV | not qualified for repeated surgery; died (suicide) after 4,5 yr |
| 28 | left | transitory hemiparesis; temporary dysphasia; full hemianopia | HS type 1; FCD IIA | Engel Class IB | follow-up 10 yr |
| 29 | right | quadrantopsia | HS type 2 | Engel Class III | reoperation; follow-up 10 years |
| 30 | left | none | HS UAC | Engel Class IB | follow-up 10 years |
| 31 | right | quadrantopsia | HS type 1 | Engel Class IA | follow-up 10 years |
| 32 | left | none | HS type 1; FCD IIA | Engel Class IB | follow-up 10 years |
| 33 | right | EPS | HS UAC | Engel Class IV | follow-up 3,5 yr |
| 34 | right | none | HS type 1; FCD IIA | Engel Class IA | follow-up 9 yr |
| 35 | left | none | HS type 1 | Engel Class IA | follow-up 9 yr |
| 36 | right | quadrantopsia | HS UAC; FCD IIA | Engel Class IA | follow-up 3 yr |
| 37 | left | none | HS type 1 | Engel Class IA | follow-up 9 yr |
| 38 | right | none | HS type 1; FCD IIA | Engel Class IV | reoperation; follow-up 9 yr |
| 39 | left | quadrantopsia | HS UAC | Engel Class II | follow-up 8,5 yr |
| 40 | right | none | HS type 1 | Engel Class II | follow-up 9 yr |
| 41 | right | none | HS type 1; FCD IIA | Engel Class IA | follow-up 8 yr |
| 42 | left | quadrantopsia | HS UAC | Engel Class IA | follow-up 3,5 yr |
| 43 | right | none | HS type 1 | Engel Class ID | follow-up 8 yr |
| 44 | right | quadrantopsia | HS type 3; FCD IIA | Engel Class IV | follow-up 8 yr |
| 45 | left | none | HS UAC | Engel Class IA | follow-up 4 yr |
| 46 | right | quadrantopsia; EPS | HS type 1; FCD IIA | Engel Class III | reoperation; follow-up 8 yr |
| 47 | left | none | HS type 2 | Engel Class IA | follow-up 8 yr |
| 48 | left | none | HS type 1 | Engel Class IA | follow-up 7 yr |
| 49 | right | none | HS UAC; FCD IIA | Engel Class IC | follow-up 7 yr |
| 50 | left | none | HS type 1 | Engel Class IA | follow-up 7 yr |
| 51 | right | quadrantopsia | HS type 1; FCD IIA | Engel Class IA | follow-up 4,5 yr |
| 52 | left | none | HS UAC | Engel Class IA | follow-up 7 yr |
| 53 | right | none | HS type 2 | Engel Class IA | follow-up 7 yr |
| 54 | left | quadrantopsia | HS UAC | Engel Class IC | follow-up 7 yr |
| 55 | right | none | HS type 1; FCD IIA | Engel Class IA | follow-up 6 yr |
| 56 | right | none | HS type 1; FCD IIA | Engel Class IB | follow-up 6 yr |
| 57 | left | none | HS type 1 | Engel Class IA | follow-up 6yr |
| 58 | right | quadrantopsia; EPS | HS type 1 | Engel Class IA | follow-up 6 yr |
| 59 | right | none | HS UAC; FCD IIB | Engel Class IV | reoperation; follow-up 6 yr |
| 60 | left | none | HS type 1 | Engel Class IA | follow-up 3,5 yr |
| 61 | right | none | HS type 1 | Engel Class IA | follow-up 6 yr |
| 62 | left | quadrantopsia | HS UAC; FCD IIA | Engel Class II | follow-up 5 yr |
| 63 | left | transitory hemiparesis; temporary dysphasia; full hemianopia | HS type 1 | Engel Class IA | follow-up 3 yr |
| 64 | right | none | HS type 1; FCD IIA | Engel Class III | not qualified for repeated surgery; follow-up 5 yr |
| 65 | left | quadrantopsia | HS UAC | Engel Class IA | follow-up 5 yr |
| 66 | right | none | HS type 1 | Engel Class II | follow-up 5 yr |
| 67 | left | none | HS type 1 | Engel Class IA | follow-up 5 yr |
| 68 | right | none | HS type 1; FCD IIA | Engel Class IV | not qualified for repeated surgery; follow-up 5 yr |
| 69 | right | none | HS type 1 | Engel Class IA | follow-up 3,5 yr |
| 70 | left | none | HS UAC; FCD IIA | Engel Class IA | follow-up 4 yr |
| 71 | left | quadrantopsia | HS type 1 | Engel Class IA | follow-up 4 yr |
| 72 | right | none | HS type 2 | Engel Class IA | follow-up 3 yr |
| 73 | left | quadrantopsia | HS UAC; FCD IIA | Engel Class IV | not qualified for repeated surgery; follow-up 4 yr |
| 74 | left | none | HS type 3 | Engel Class IC | follow-up 4 yr |
| 75 | right | none | HS UAC | Engel Class IA | follow-up 4 yr |
| 76 | left | EPS | HS type 1; FCD IIA | Engel Class IV | not qualified for repeated surgery; follow-up 3 yr |
| 77 | right | none | HS type 1 | Engel Class IA | follow-up 3 yr |
| 78 | right | none | HS type 2; FCD IIA | Engel Class IA | died (cancer); follow-up 3 yr |
| 79 | right | full hemianopia | HS UAC | Engel Class IA | follow-up 3 yr |
| 80 | left | none | HS type 1 | Engel Class IA | follow-up 3 yr |
| 81 | right | none | HS type 1 | Engel Class IA | follow-up 2,5 yr |
| 82 | left | none | HS UAC; FCD IIA | Engel Class IA | follow-up 3 yr |

HS – hippocampal sclerosis; FCD – focal cortical dysplasia; UAC – unable to classify; EPS – early postoperative seizures
